# Probing the physical properties for prospective high energy applications of QMnF_3_ (Q = Ga, In) halide perovskites compounds employing the framework of density functional theory

**DOI:** 10.1039/d3ra02878j

**Published:** 2023-06-20

**Authors:** Fareesa Tasneem Tahir, Mudasser Husain, Nourreddine Sfina, Ahmed Azzouz Rached, Majid Khan, Nasir Rahman

**Affiliations:** a Department of Physics, Abdul Wali Khan University Mardan KPK Pakistan majidkhan@awkum.edu.pk; b Department of Physics, University of Lakki Marwat 28420 Lakki Marwat KPK Pakistan nasir@ulm.edu.pk; c College of Sciences and Arts in Mahayel Asir, Department of Physics, King Khalid University Abha Saudi Arabia; d Magnetic Materials Laboratory, Faculty of Exact Sciences, Djillali Liabes University of Sidi Bel-Abbes Algeria

## Abstract

We use WIEN2K to conduct density functional theory computations to explore the structural, thermodynamic, optoelectronic, and mechanical properties of fluoroperovskites QMnF_3_ (Q = Ga, In). The application of the Birch–Murnaghan equation to the energy *versus* volume, formation energy, and tolerance factor confirms the structural stability of these two QMnF_3_ (Q = Ga, In) materials. The thermodynamic stability of the compounds is confirmed by the results of the phonon calculation, while the mechanical stability is confirmed from the values of the elastic constants. GaMnF_3_ demonstrates a high capacity to withstand both compressive and shear stresses. A lower bulk modulus is responsible for the weaker ability of InMnF_3_ to endure changes in volume. Compared to GaMnF_3_, InMnF_3_ possesses rigidity having greater shear modulus, indicating greater resistance to changes in shape. However, both compounds are characterized as mechanically brittle, anisotropic, and ductile. The band structure that was determined indicates that both GaMnF_3_ and InMnF_3_ exhibit a metallic character. The density of states analysis further supports the metallic nature of GaMnF_3_ and InMnF_3_. In GaMnF_3_, the “Mn” and “F” atoms in the valence band significantly participate in the total density of states, whereas in InMnF_3_, both “Mn” and “F” atoms also dominate the total density of states. The values of *ε*_1_(0) computed for GaMnF_3_ and InMnF_3_ are positive *i.e.* > 0, and agree with Penn's model. We calculate the optical properties for both GaMnF_3_ and InMnF_3_ and the potential of these materials of interest for applications in optoelectronic gadgets including light-emitting diodes is attributed to their absorption in the ultraviolet-visible zone. We believe that this work may provide comprehensive insight, encouraging further exploration of experimental studies.

## Abstract 2

We use WIEN2K to conduct density functional theory computations to explore the structural, thermodynamic, optoelectronic, and mechanical properties of fluoroperovskites QMnF_3_ (Q = Ga, In).

## Graphical abstract

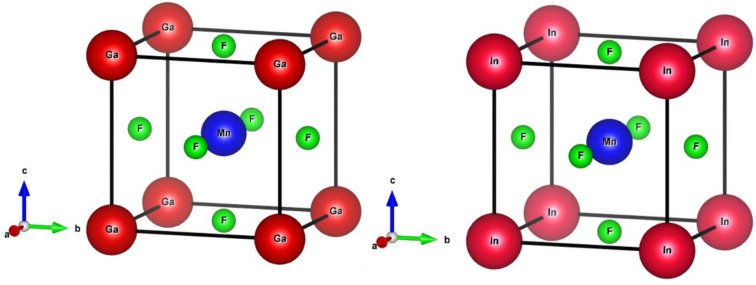

## 1. Introduction

The recent attention given to cubic fluoroperovskites (ABX_3_), with “A” and “B” representing cations, is attributed to their favorable crystalline structures and promising physical properties. Nishimatsu *et al.*^1^ conducted a DFT (density functional theory) analysis of various ABX_3_ compounds to determine their band structure by employing the pseudo-potential method and local density approximation (LDA).^2^ Materials scientists have worked on perovskite materials for various technological applications.^3–8^ The Trans-Blaha-mBJ approximation is utilized to analyze the optoelectronic properties of SrTiO_3_ and BaTiO_3_ at room temperature, and the findings surpassed the outcomes of previous *ab initio* investigations in terms of measured values.^9^ The structural, electronic, and optical properties of ACaF_3_ (A = K, Rb, Cs) were computed to predict their potential use in the optoelectronics industry and to identify which material possesses a wide and direct band gap. This analysis aimed to determine the bonding nature of the compounds as well.^10–13^ By employing GGA (generalized gradient approximation) and GGA + U methods, researchers analyzed the optical properties of several systems including rare earth.^14–17^ It was found that the TB-mBJ approximation of the TB-mBJ potential is better suited for systems lacking correlated electrons, such as insulators and semiconductors. The TB-mBJ approximation was deemed superior not only regarding the results but concerning computational efficiency as well. Murtaza *et al.*^18^ employed the TB-mBJ method to compute the electrical and optical properties and explain the reduction in bulk modulus and the rise in crystal lattice constants. Solid solutions of LiKBaMgF_3_ on LiBaF_3_ were synthesized for their potential usage in the deep ultraviolet (DUV) range.^19^ Studzinski *et al.*^20^ conducted experimental research on structural phase transitions in RbCdF_3_. Studies have been executed on the cubic fluoroperovskites ACaF_3_ (A = Rb, K, and Cs) as potential materials suitable for luminescence.^21–24^ Three factors are crucial for core-valence luminescence (CVL), including (1) the energy gap within the p-states of “F” and the core p-states of “Rb, K, and Cs”, comprising the upper region of the valence band; (2) the breadth of the higher portion of the valence band (3) band gap. These investigations, utilizing the *ab initio* approach, reignited interest in these systems. Various researchers^25–27^ researched the optical properties of cubic perovskites, employing a full potential linearized augmented plane wave (FP-LAPW) approach with GGA and LDA. The scientists examined the temperature-dependent structural characteristics and phase transitions of RbCaF_3_ to propose an order–disorder behavior for the phase transitions at 193 K and 50 K.^28^ Nonetheless, the structural, optical, phononic, mechanical, and electronic properties of the cubic perovskites QMnF_3_ (where Q = Ga, In) have yet to be investigated or established by the TB-mBJ method.

The utilization of FP-LAPW within the DFT framework was prompted by the need for more accurate optical properties that can effectively address band gaps and transitions, which are expected to be provided by mBJ-based computations. To gain a deeper understanding of the stability of these structures, their thermodynamic properties have also been analyzed.

## 2. Computational details and crystal structure

**Fig. 1:**
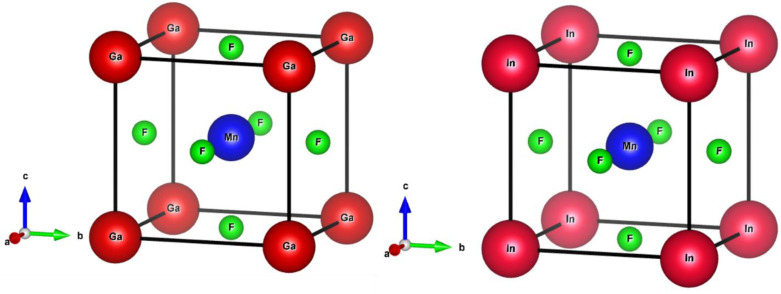
Crystalline cubic structure of QMnF_3_ (Q = Ga, In) fluoroperovskites compounds.

The ternary compounds QMnF_3_ (where Q = Ga, In) crystallizes in cubic symmetry having *Pm*3̄*m* space group, as illustrated in Fig. 1. Cubic symmetry is a fascinating aspect of symmetry found in various objects and materials. Its regular and highly symmetrical arrangement gives rise to unique properties and behaviors. Understanding cubic symmetry is essential for many scientific disciplines and has practical applications in materials science, electronics, optics, and more.

Based on the Wyckoff coordinates, the Q (Ga, In), Mn, and fluorine atoms are situated at positions (0, 0, 0), (0.5, 0.5, 0.5), and (0, 0.5, 0.5), correspondingly.^29^ DFT is a quantum mechanical and firmly established theory used for solving many-body problems.^30^ In this study, the total energy computation by first-principles approach^31,32^ was performed using FP-LAPW based on the DFT approach implemented in the WIEN2k package,^33^ which is an effective technique for calculating the ground-state properties of materials. The primitive unit cell is split into two parts: (I) the interstitial zone and (II) non-overlapping centered atomic spheres on atomic sites. Distinct basis sets are utilized in each of these two areas. In GaMnF_3_, the muffin-tin radii (RMT) for the “Ga, Mn, and F” atoms have been set to 2.47, 1.54, and 1.78 a.u., respectively. Furthermore, for InMnF_3_, the muffin tin (MT) radii of the In, Mn, and F atoms are set at 2.52, 1.61, and 1.92 a.u., respectively. The convergence parameters, with a value of *R*_MT_ × *K*_MAX_ = 8, were used to control the magnitude of the basis sets composed of plane waves. To maintain consistency, the value of G_MAX_ is fixed at 11 (a.u.)^−1^, convergence is attained when the energy tolerance reaches 10^−3^ Ry. The electronic structure in the ground state of the compound under investigation is calculated using the lattice parameters having an ideal value. To establish the ideal value of the lattice parameters, we computed the total energies for a range of volumes that extend from −10% to +10% of the measure (or theoretical) lattice constants.^34,35^ The investigated outcomes are presented graphically using equations provided in the ref. 36. The TB-mBJ method has been successful in improving the accuracy of band gap results for semiconductors,^37^ as well as in computing optical properties, as demonstrated in the present study using the FP-LAPW approach. The IRelast package^38^ was utilized to calculate various elastic property parameters.

## 3. Results and discussion

### 3.1. Structural and phonon properties

**Fig. 2:**
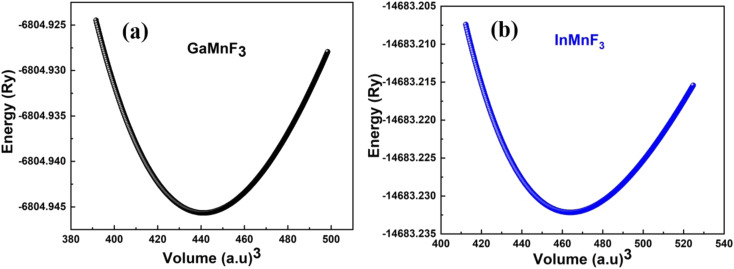
Optimization curves of energy *versus* volume for (a) GaMnF_3_ (b) InMnF_3_ ternary fluoroperovskites compounds.

Fig. 2a and b illustrate the optimal energy curves of cubic GaMnF_3_ and InMnF_3_ ternary fluoroperovskites, fitted using the Birch–Murnaghan equation.

These computations predict various properties, including the ground-state energy (*E*_0_), ground-state volume (*V*_0_), bulk modulus (*B*_0_), and pressure derivative 
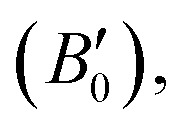
 of the structure. The parameters are regarded as *E*_0_ and *V*_0_.^39–42^ To determine the materials' ground state, one can examine the points on the curve where the Birch–Murnaghan equation is fitted to the lowest energy concerning volume. The determination of the *V*_0_ can be employed to compute lattice constants. Aside from optimizing the volume through the Birch–Murnaghan EOS (equation of states), the thermodynamic stability is also assessed by calculating the formation energy (*H*_f_) of both compounds using eqn (1).

1 *H*_f_ = *E*_QMnF_3__ − (*aE*_Q_ + *bE*_Mn_ + *cE*_F_)

Eqn (1) involves the compound total energy *E*_QMnF_3__ as well as the individual energies of Q (Ga, In), Mn, and F, denoted as *E*_Q_, *E*_Mn_, and *E*_F_, respectively. The computed formation energies of GaMnF_3_ and InMnF_3_ are −2.35 Ry and −11 321.92 Ry, respectively. The compounds' negative formation energies indicate that they are thermodynamically stable, with GaMnF_3_ displaying greater thermodynamic stability than InMnF_3_. Table 1 summarizes the structural parameters of fluoroperovskites GaMnF_3_ and InMnF_3_.

**Table 1:** Structural parameters of both GaMnF_3_ and InMnF_3_ optimized through the fitted curves of Birch–Murnaghan EOS

Optimized structural parameters	GaMnF_3_	InMnF_3_
*a*_0_ in Å	4.02	4.09
*E*_0_ in Ry	−6804.94	−14 683.23
*V*_0_ in (a.u.)^3^	441.14	466.23
*B* in GPa	103.33	84.58
*B*′ in GPa	2.52	4.02
Δ*H*_f_ (eV)	−2.35	−11 321.92

#### 3.1.1. Phonon band structures and density of states

**Fig. 3:**
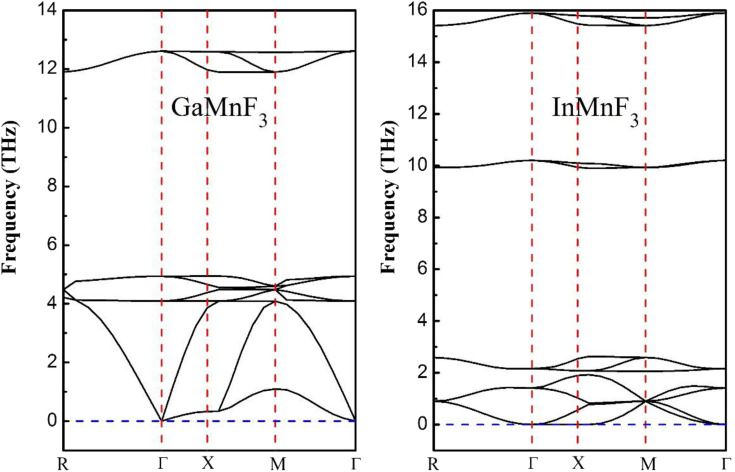
Phonon band dispersions curves of frequency *vs.* momentum for ternary GaMnF_3_ and InMnF_3_ fluoroperovskites.

**Fig. 4:**
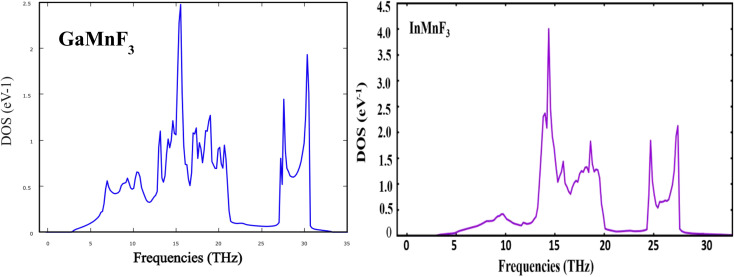
Phonons density of states for GaMnF_3_ and InMnF_3_ ternary fluoroperovskites compounds.

The phonon band structure is usually represented as a plot of the phonon frequency as a function of the wave vector. The frequency is typically plotted along the vertical axis, and the wave vector (or its components) is plotted along the horizontal axis. The resulting graph shows the allowed energy levels or bands corresponding to different phonon modes. Phonon band structures provide valuable insights into the vibrational properties of materials. They can help understand phenomena like lattice dynamics, thermal transport, and phonon–electron interactions. Additionally, phonon band structures are crucial for studying the thermal and mechanical properties of materials and for designing new materials with specific phononic properties. The absence of imaginary frequencies in Fig. 3 and 4 obtained from the computation of phonon properties, confirms the thermodynamic stability. The phonon DOS shown in Fig. 4 is typically represented as a plot of the number of phonon states *versus* frequency. The phonon DOS is calculated theoretically using the techniques, such as density functional theory (DFT). The phonon DOS is closely related to the phonon band structure. The phonon DOS can be obtained from the phonon band structure by integrating the density of states overall wave vectors or energy bands. It is very obvious from the figures of phonon band structure and phonon density of states that no states exist at negative frequency ranges and thus confirming the thermodynamic stability.

Both GaMnF_3_ and InMnF_3_ exhibit only real frequencies, indicating that no imaginary frequencies are present. The fact that both compounds exhibit only real frequencies suggests that they are thermodynamically stable. Consequently, the confirmation of both negative formation energy and thermodynamic stability indicates that both materials possess structural stability.

### 3.2. Mechanical properties

The elastic properties of the compounds were assessed to analyze their mechanical stability. This was accomplished by determining the elastic constants, which reveal the reaction of the material to the forces applied and thus its mechanical characteristics. Therefore, these constants offer insights into the mechanical stability and resilience of the materials. The stress-tensor components for small strains were investigated at 0 GPa pressure, and the energy is divided by the lattice strain to maintain constant volume. To calculate the elastic constants, we utilized the IRelast software package, designed for cubic crystalline symmetry and integrated into WIEN2K. For cubic structures, there are only three independent elastic constants, which are *C*_11_, *C*_12_, and *C*_44_. Table 2 presents several elastic parameters for the materials, which were computed using the equations outlined in the study conducted by Khattak *et al.*^43^ In cubic crystal structures, the elastic constants must be interdependent for mechanical stability, satisfying the following conditions: *C*_11_ − *C*_12_ > 0, *C*_11_ > 0, *C*_44_ > 0, *C*_11_ + 2*C*_12_ > 0, and *B* > 0.^44,45^Table 2 indicates that the investigated materials satisfy these criteria, indicating their mechanical stability and hardness. As shown in Table 2, GaMnF_3_ exhibits higher mechanical stiffness coefficients, indicating that interested materials can withstand both compressive and shear stresses effectively.^46^ In contrast, InMnF_3_ displays a lower B (bulk modulus), suggesting a relatively weaker ability to bear volume changes. Moreover, the higher *G*-modulus of rigidity in InMnF_3_ compared to GaMnF_3_ implies that InMnF_3_ exhibits greater resistance to shape deformation. The values of “A” indicate that none of the compounds are entirely isotropic. Notably, the *B*/*G* ratio of the materials is negative, signifying the ductile nature of both compounds.

**Table 2:** Elastic parameters for GaMnF_3_ and InMnF_3_ including the ECs (elastic constants) (*C*_11_, *C*_12_, and *C*_44_), bulk modulus (*B*), anisotropy factor *A*, Poisson's ratio ν, Pugh ratio (*B*/*G*), machinability index (*μ*_M_), Cauchy pressure (*C*_11_–*C*_44_), and shear modulus (*G*)

Elastic parameters	GaMnF_3_	InMnF_3_
*C*_11_ (GPa)	162.57	147.83
*C*_12_ (GPa)	57.48	53.17
*C*_44_ (GPa)	2.05	24.78
*B* (GPa)	103.33	84.58
*A*	0.04	0.05
*E* (GPa)	18.52	18.62
*ν*	0.48	0.46
*B*/*G*	8.64	5.04
*μ*_M_	50.50	3.41
*C*_11_–*C*_12_ (GPa)	109.09	90.66
*G* (GPa)	11.96	16.77
*C*_11_–*C*_44_ (GPa)	160.52	123.05

All elastic stiffness coefficients, as well as the other constants and pressure, are given in GPa, while volume is in Bohr.

### 3.3. Electronic band structure and density of states

**Fig. 5:**
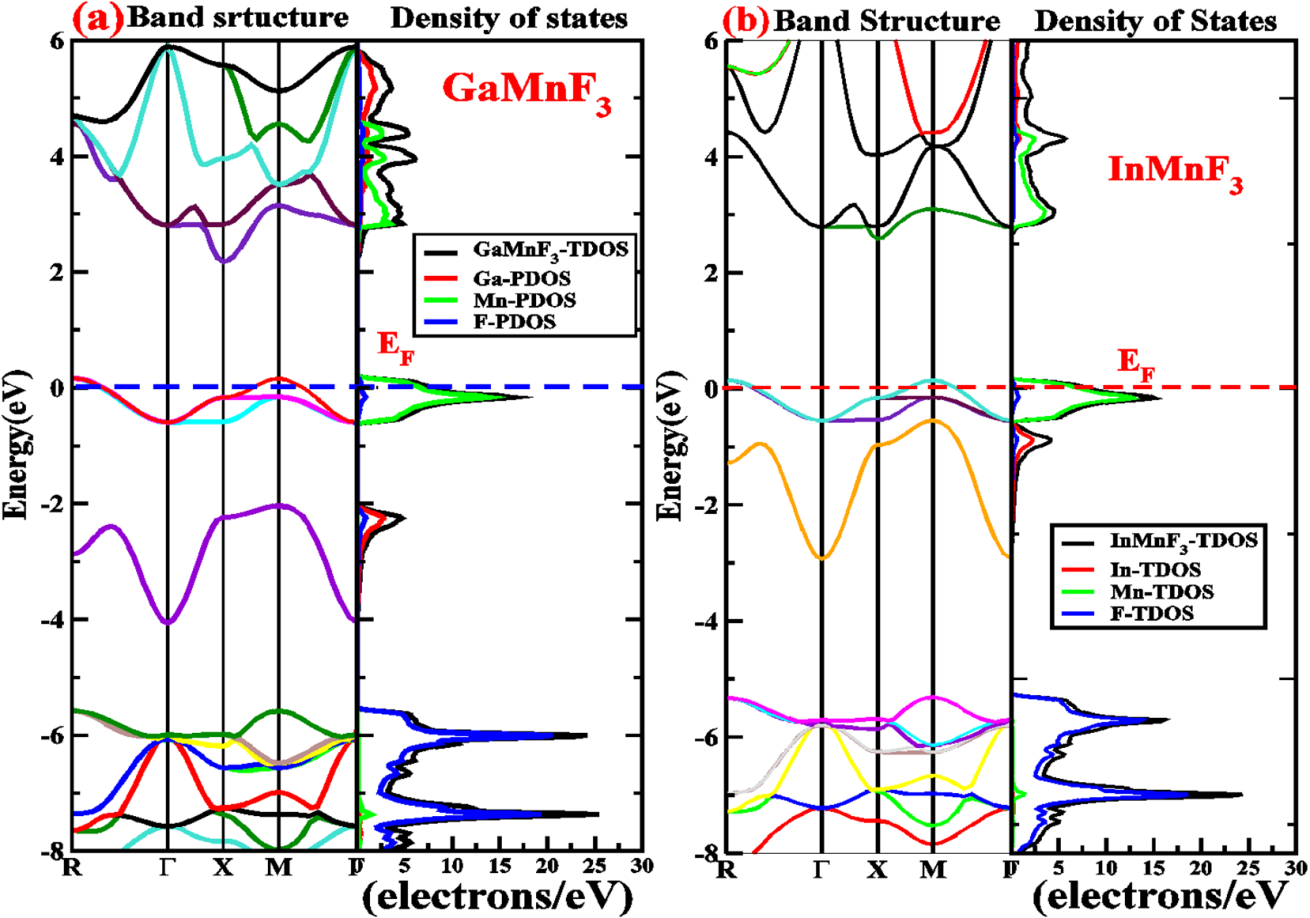
Band structures fitted with DOS of (a) GaMnF_3_ and (b) InMnF_3_ ternary fluoroperovskites compounds.

Electronic properties play a crucial impact in determining the industrial applications of materials and also significantly influence their physical properties. The methods for investigating electronic properties have been mainly introduced over the past few decades due to advancements in programming techniques, computing power, and the development of density functional theory (DFT). There are several DFT-based computational methods available to accurately determine a material's electronic structure. To analyze the electronic properties, computations (self-consistent field) have been performed.^47–50^ The main determinants of a material's electronic characteristics are its band structure and density of states (DOS). Using the potential TB-mBJ approximation and the design of an optimized unit cell, the electronic properties of selected materials are computed by performing calculations at the high-symmetry points of the Brillouin zone. The electronic band structures of the fluoroperovskites GaMnF_3_ [Fig. 5a] and InMnF_3_ [Fig. 5b] were calculated, and the outcomes are displayed in Fig. 5. Based on the results shown in the figures it can be inferred that the conduction band minima and valence band maxima overlap around the Fermi level, indicating metallic behavior for both materials.

**Fig. 6:**
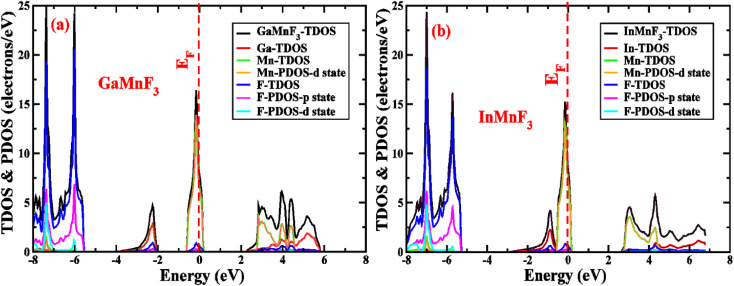
The total density of states and partial density of states (a) GaMnF_3_ (b) InMnF_3_ ternary fluoroperovskites compounds.

To gain more understanding of the electrical properties of the investigated structures, we also calculated the DOS, as shown in Fig. 6. The metallic nature of GaMnF_3_ and InMnF_3_ fluoroperovskites is further confirmed by the total density of states (TDOS & PDOS) shown in Fig. 6a and b, respectively, which agree with the results achieved from the band structures. The TDOS and PDOS of GaMnF_3_ are primarily influenced by fluorine in the VB energy range [within −10 eV and −5 eV] and [between −5 eV and 0 eV] near Fermi level and are slightly overlapping in the conduction band, while the participation to the TDOS in the CB is dominated by “Mn” atom. The dominant contribution of the “Mn” atom near the Fermi energy level confirms the metallic behavior of both compounds. On the other hand, in the case of InMnF_3_, the “F” atom is dominant in the valence band around the Fermi level and is slightly overlapped and in the conduction band, the major contributions to the DOS also comes from the “F” atom. In the context of electronic structure calculations, PDOS and TDOS are related concepts that describe the density of states of a material. The TDOS is the sum of all the electronic states of a material, regardless of their origin. It designates the total number of available electronic energy states per unit volume as a function of energy. The PDOS, on the other hand, describes the contribution of specific atoms, orbitals, or other subspaces to the overall TDOS. It provides information about the electronic structure of a material on a more localized level, allowing researchers to examine the electronic properties of specific parts of the material. The PDOS is a modification of the DOS, where the density of states is projected onto specific atoms, orbitals, or other subspaces of the material. The PDOS can be calculated using various computational methods, such as DFT. The PDOS is a useful tool for understanding the electronic structure of materials and can provide insights into their physical and chemical properties. It could be utilized to study a wide range of materials, including metals, semiconductors, and insulators, and can help researchers design new materials with specific electronic properties.

### 3.4. Optical properties

**Fig. 7:**
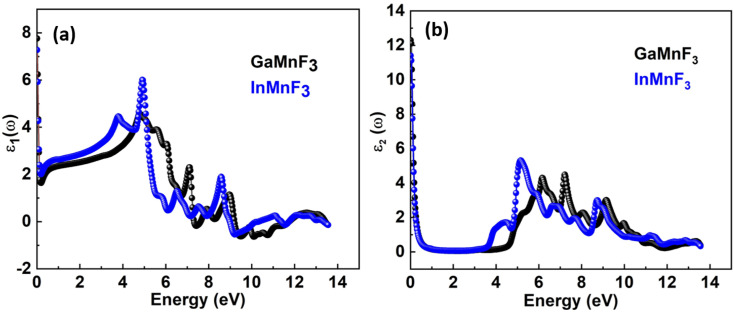
Real (a) and imaginary (b) parts of the dielectric function of QMnF_3_ (where Q = Ga, In) fluoro-perovskites.

To investigate the complex dielectric function, one must explore the investigations of GaMnF_3_ in the IR (infrared) zone at 12.39 μm, as well as the reflectivity and transmission of InMnF_3_, which demonstrates metallic behavior. The utilization of complex dielectric functions can provide insights into the behavior of solids in the presence of electromagnetic waves (EMs) and the interactions between electrons and phonons. This information can also aid in understanding the propagation of EM waves through various media. Kramer–Kronig transformation is employed for extracting the energy storage in any medium from the imaginary component of the dielectric function, by *ε*_1_(*ω*) which represents the energy stored in the medium.^51,52^ Evidence about absorption behavior, band topologies, and charge carrier transitions from filled to unoccupied states can also be obtained from *ε*_2_(ω).^53–55^Fig. 7a and b Illustrates the investigation materials' dielectric function's real and imaginary components.

**Fig. 8:**
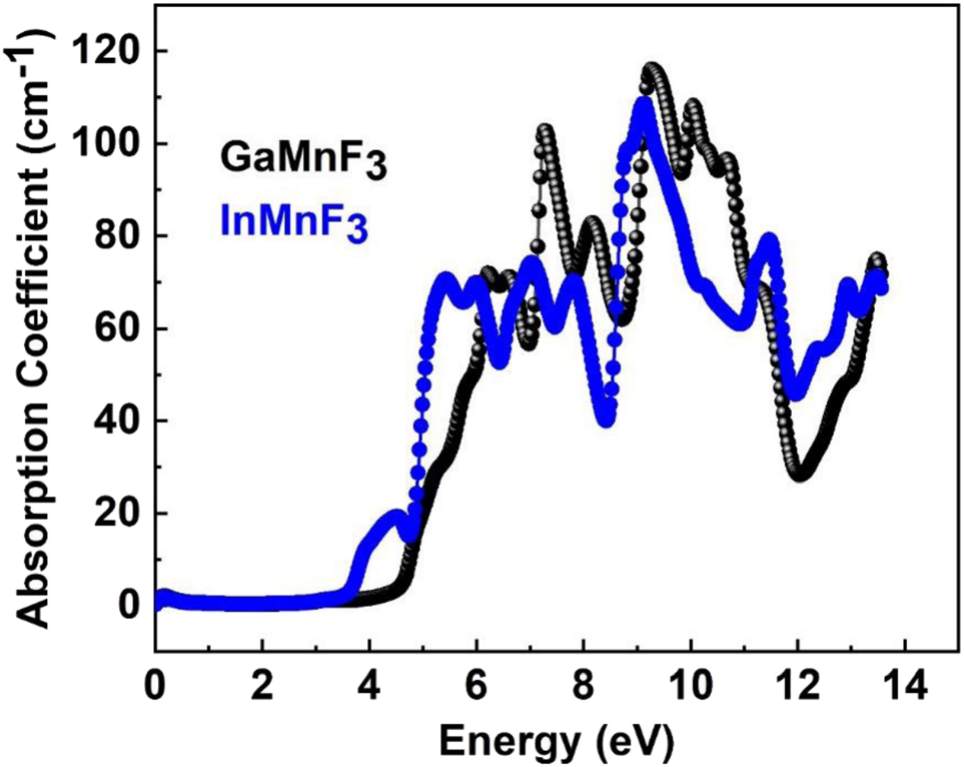
The absorption coefficient for the QMnF_3_ (where Q = Ga, In) fluoro-perovskites.

The optical responses of a material can be determined by utilizing *ε*(*ω*).^56^ The real part of the dielectric function describes the response of a material to an applied electric field and is a fundamental property used to understand the optical and electronic behavior of materials. The value of the zero-frequency limit *ε*_1_(0) is a precise indicator of *ε*_1_(*ω*), as it primarily relies on the band-gap value and denotes the electronic component of the static dielectric function. The GaMnF_3_ and InMnF_3_ exhibit values of 7.5 and 6.8, respectively, for *ε*_1_(0). The maximum value of *ε*_1_(0) in the GaMnF_3_ material is reached at 0.003 eV, and beyond that point, it gradually decreases. Conversely, InMnF_3_ exhibits a peak value for *ε*_1_ (*ω*) at 0.028 eV, after which it declines and becomes negative. The real part of the dielectric function for fluoroperovskites can become negative within a certain range of frequencies, indicating the presence of negative permittivity. This behavior has important implications for the optical properties and potential applications of these materials. It can be observed that both GaMnF_3_ and InMnF_3_ is conductive for incident photon at lower energy ranges, whereas, behaves as an insulator for high energies of incident photons (as indicated by the negative values of *ε*_1_(*ω*)). The graph (Fig. 7b) illustrates the behavior of the imaginary component of the dielectric constant for the mentioned compounds. The photon absorption-related factor *ε*_2_(*ω*) is widely recognized as the primary determinant of a crystalline material's electrical behavior. As depicted in Fig. 7b, the *ε*_2_(*ω*) spectra for GaMnF_3_ and InMnF_3_ exhibit an initial peak at 0.007 and 0.085, respectively, followed by a gradual decline as energy levels increase. These peaks signify the transition from the VB to the CB. Moreover, as the dielectric function of each studied compound varies in different directions, their real and imaginary components demonstrate their anisotropic nature. The absorption coefficient is an important variable utilized to quantify the absorption of light. The spectrum of a material can be used to measure the attenuation of light intensity and distance as it passes through the material, and its behavior can be foreseen by examining its dielectric function. Fig. 8 depicts the investigated spectra of the absorption coefficient for selected materials, which reveals the absence of absorption peaks within the visible or infrared (IR) energy range. This suggests that ternary GaMnF_3_ and InMnF_3_ compounds are transparent within this region.

**Fig. 9:**
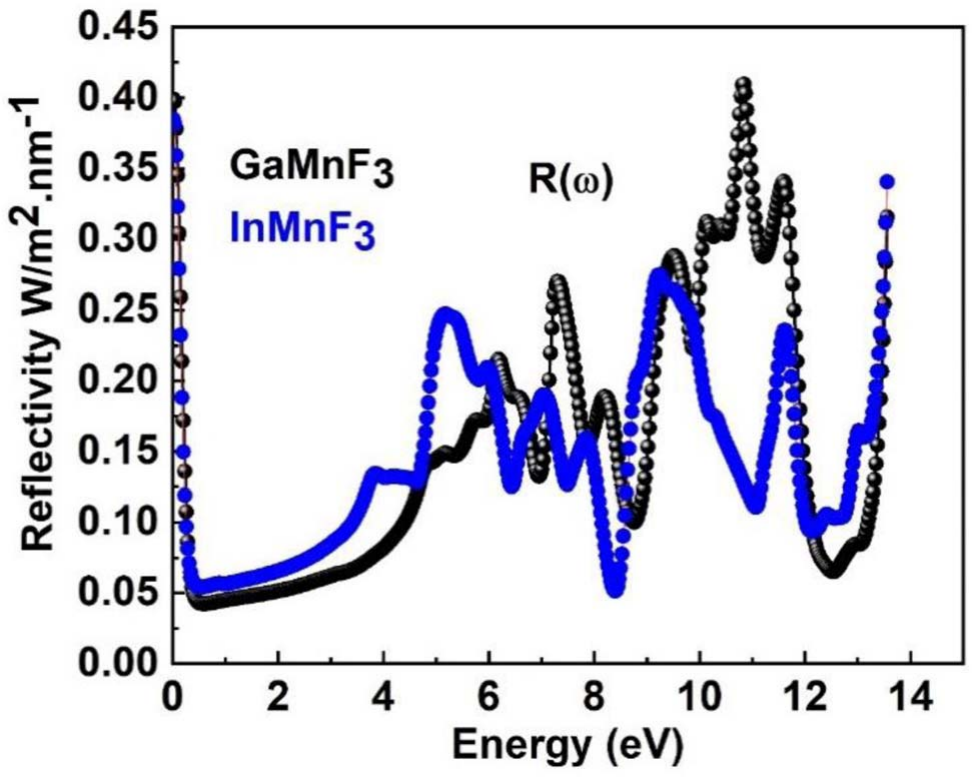
The reflectivity of the QMnF_3_ (where Q = Ga, In) fluoro-perovskites.

The maximum absorption peaks within the UV energy zone occur at 9.27 eV and 9.11 eV for GaMnF_3_ and InMnF_3_, respectively. Intense light absorption occurs in the UV region for both materials. The inter-band transition of electrons leads to the generation of absorption peaks. These materials have the potential for optoelectronic applications because they exhibit no light absorption within visible spectra. The reflectivity expressed by *R*(*ω*), is a critical parameter for evaluating the suitability of a material for shielding applications, especially as an antireflective coating. Fig. 9 displays the spectra of reflectivity for ternary GaMnF_3_ and InMnF_3_ materials being studied, where GaMnF_3_ and InMnF_3_ at the static level exhibit the values of *R*(*ω*) to be 0.39 and 0.38, respectively.

**Fig. 10:**
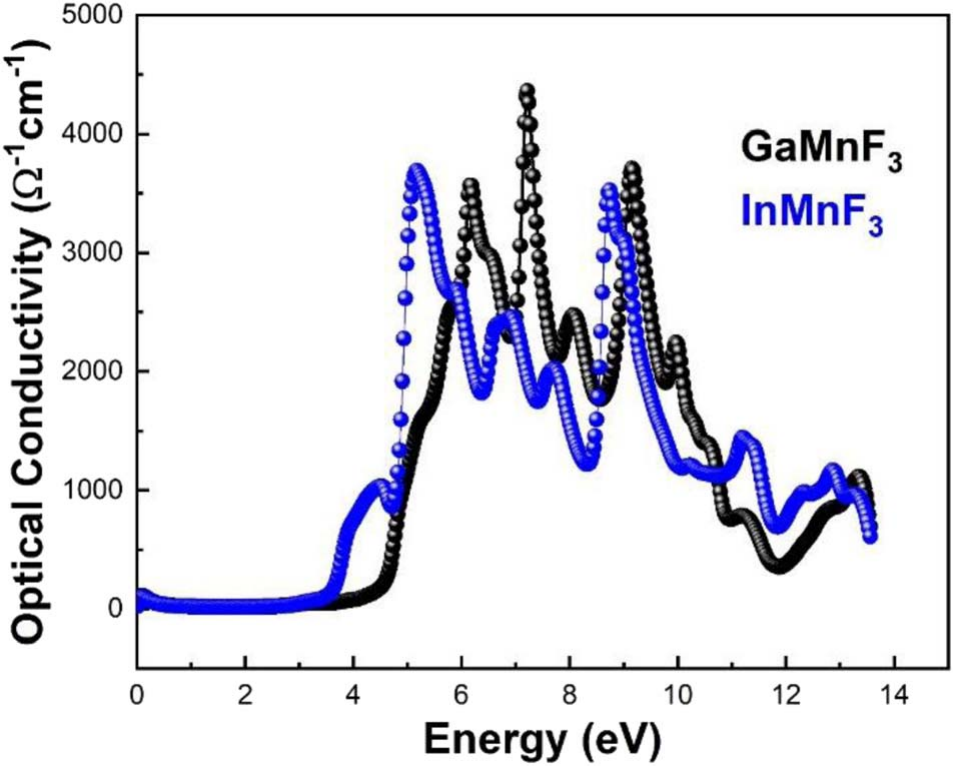
Optical conductivity of ternary QMnF_3_ (where Q = Ga, In) fluoroperovskites.

The reflectance of both the compounds shows a sharp decline after the static level, followed by a gradual increase until it reaches its maximum at approximately 0.39 eV and 0.38 eV for GaMnF_3_ and InMnF_3_, respectively, followed by dramatic fluctuations (increase and decreased) in the energy range from 4 eV to 14 eV. Optical conductivity is a measure of the response of a material's induced current density to an externally applied electric field at a specific frequency. The optical conductivity spectra for the two ternary QMnF_3_ (where Q = Ga, In) fluoroperovskites are displayed in Fig. 10, illustrating their behavior for incident photon energies ranging from 0 eV to 14 eV.

**Fig. 11:**
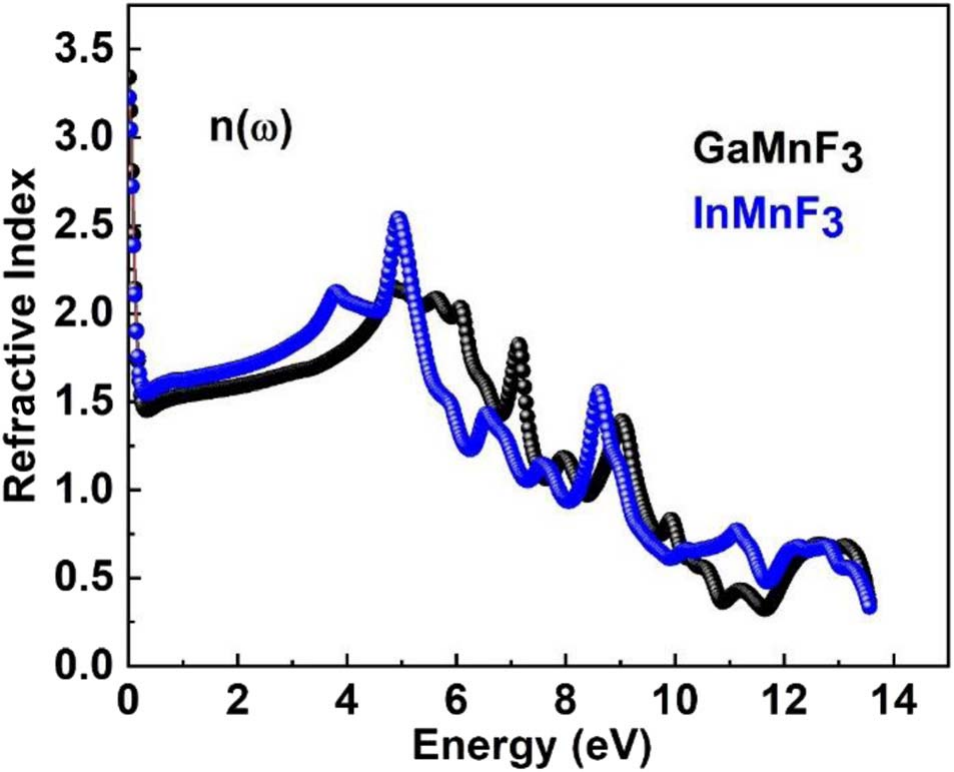
Refractive index for ternary QMnF_3_ (where Q = Ga, In) fluoro-perovskites.

At an incident photon energy of 7.19 eV, GaMnF_3_ displays a maximum optical conductivity of 4347.90 Ohm^−1^ cm^−1^, while InMnF_3_ displays a maximum optical conductivity of 3711.37 Ohm^−1^ cm^−1^ at 5.15 eV incident photon energy, as demonstrated in Fig. 10. The optical conductivity of both ternary QMnF_3_ (where Q = Ga, In) fluoroperovskites is high at low energies, indicating that these materials are suitable for use in optical devices that operate in the UV-vis range. Fig. 11 displays the refractive index calculated for GaMnF_3_ and InMnF_3_ in the energy range of 0 to 14 eV.

**Fig. 12:**
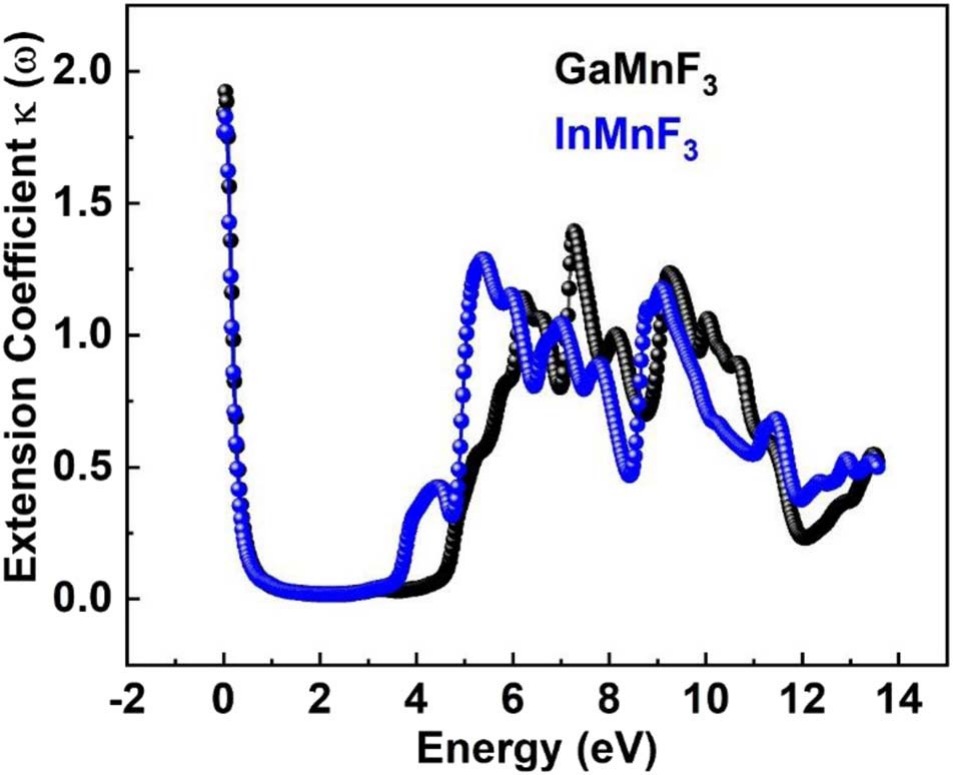
Extension Coefficient for ternary QMnF_3_ (where Q = Ga, In) fluoroperovskites compounds.

The values for GaMnF_3_ and InMnF_3_ are 3.34 and 3.32, respectively, at lower energies of 0.034 and 0.007 eV. The curves for both materials intersect and decrease as energy increases, and then fluctuates dramatically for the remaining energy range. The difference in refractive index between GaMnF_3_ and InMnF_3_ indicates that the electronic polarization in the medium of GaMnF_3_ causes a greater delay in the passage of photons compared to InMnF_3_. The electronic polarization, which affects the speed of photons passing through a medium and ultimately determines the refractive index, is influenced by the atomic size that composes the material. Since the In atom is larger, it generates a more significant polarization effect, leading to a higher degree of retardation (*i.e.*, slower photon velocity) and a lower refractive index. At 4.7 and 4.9 eV, the refractive index start decreasing followed by dramatic fluctuations in the energy range from 4.5 to 14 eV. The purpose of deriving the extinction coefficient, *k* (*ω*), was to demonstrate the degree to which a material absorbs light, shown in Fig. 12.

**Fig. 13:**
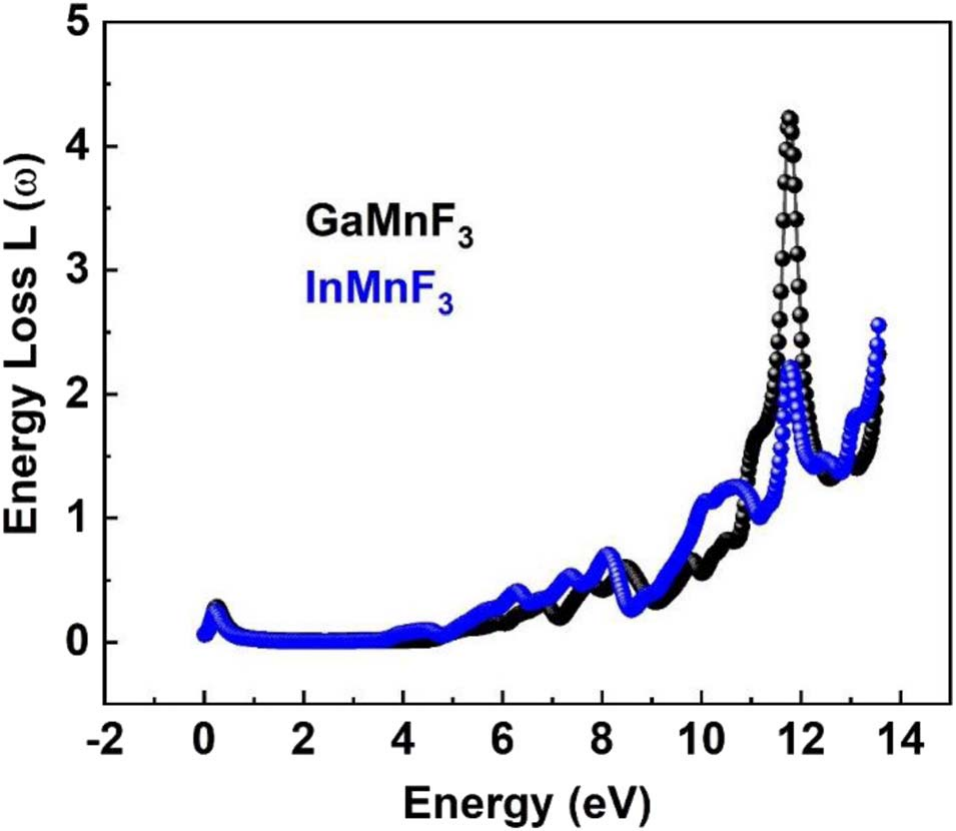
The energy loss function for ternary QMnF_3_ (where Q = Ga, In) fluoro-perovskites.

At 0.007 eV, the extinction coefficient for GaMnF_3_ is at its highest value of 1.92, as depicted by the black-colored data in Fig. 10. Similarly, the maximum value of the extinction coefficient for InMnF_3_ is 1.82, as represented by the blue-colored data in the same figure, and occurs at 0.085 eV. In both materials, the extinction coefficient shows a sharp decrease and fluctuates slightly as energy levels increase. When an electron travels at high speed through a substance, it experiences a loss of energy. Various phenomena can result from this energy loss, such as the excitation of phonons and Plasmon, inter-band transitions, and ionization of inner shells.^57^ The plasma frequency occurs at the point where the energy loss function (ELF) touches its maximum value.^58^ Based on the ELF spectra depicted in Fig. 13, the static level of all compounds indicates zero energy loss, while the peak energy loss for GaMnF_3_ commences at 11.78 eV, and for InMnF_3_, it seems at 11.81 eV.

For both materials, a significant increase in energy loss values is observed within the higher 10 eV to 14 eV energy range.

Chloroperovskites, specifically referring to hybrid organic-inorganic perovskite materials that contain chlorine, have gained significant attention in the field of solar energy and optoelectronics. These materials exhibit excellent photovoltaic and optoelectronic properties, making them suitable for various high-energy applications which include solar cells, photo-detectors, Light-Emitting Diodes (LEDs), X-ray detectors, and lasers. It's worth noting that the field of perovskite materials, including chloroperovskites, is still undergoing extensive research and development. While the performance and stability of perovskite-based devices have significantly improved, there are ongoing efforts to address the challenges related to long-term stability, scalability, and commercial viability in practical applications.

## 4. Conclusion

In conclusion, the physical properties of fluoroperovskites QMnF_3_ (where Q = Ga, In) which includes the structural and thermodynamic stability, phononic, optoelectronic, and mechanical properties were examined using the density functional theory implemented in WIEN2K. The data fitted through Birch–Murnaghan EOS for the energy *vs.* volume corroborated the fluoroperovskites structural stability, and the negative values of formation energy and phonon dispersion spectra for interested compounds demonstrate the thermodynamic stability. The elastic constants demonstrated a high capacity to withstand compressive and shear stresses due to their superior elastic stiffness coefficients. InMnF_3_ demonstrated a lower ability to withstand changes in volume owing to its lower bulk modulus, indicating a weaker resistance to compression. However, it exhibited a higher *G*-modulus of rigidity than GaMnF_3_, suggesting a greater strength in resisting changes in shape. Anisotropy and brittleness were observed in both compounds. The metallic nature of GaMnF_3_ and InMnF_3_ was determined by analyzing their band gaps. In addition, the density of states study confirmed the metallic nature of GaMnF_3_ and InMnF_3_. The valence band of GaMnF_3_ exhibited significant contributions from Ga and F atoms, whereas the TDOS of InMnF_3_ is mainly participated by “Mn” and “F” atoms. The calculated values of *ε*_1_(0) for GaMnF_3_ and InMnF_3_ are consistent with Penn's model. The absence of absorption in the visible range confirmed that the investigated materials are strong candidates for optoelectronics applications.

## Conflicts of interest

There are no conflicts to declare.

## Supplementary Material
